# Characterizing Adults Receiving Primary Medical Care in New York City: Implications for Using Electronic Health Records for Chronic Disease Surveillance

**DOI:** 10.5888/pcd13.150500

**Published:** 2016-04-28

**Authors:** Matthew L. Romo, Pui Ying Chan, Elizabeth Lurie-Moroni, Sharon E. Perlman, Remle Newton-Dame, Lorna E. Thorpe, Katharine H. McVeigh

**Affiliations:** Author Affiliations: Matthew L. Romo, New York City Department of Health and Mental Hygiene, Long Island City, New York, and City University of New York School of Public Health, New York, New York; Elizabeth Lurie-Moroni, Sharon E. Perlman, Remle Newton-Dame, Katharine H. McVeigh, New York City Department of Health and Mental Hygiene, Long Island City, New York; Lorna E. Thorpe, City University of New York School of Public Health, New York, New York.

## Abstract

**Introduction:**

Electronic health records (EHRs) from primary care providers can be used for chronic disease surveillance; however, EHR-based prevalence estimates may be biased toward people who seek care. This study sought to describe the characteristics of an in-care population and compare them with those of a not-in-care population to inform interpretation of EHR data.

**Methods:**

We used data from the 2013–2014 New York City Health and Nutrition Examination Survey (NYC HANES), considered the gold standard for estimating disease prevalence, and the 2013 Community Health Survey, and classified participants as in care or not in care, on the basis of their report of seeing a health care provider in the previous year. We used χ^2^ tests to compare the distribution of demographic characteristics, health care coverage and access, and chronic conditions between the 2 populations.

**Results:**

According to the Community Health Survey, approximately 4.1 million (71.7%) adults aged 20 or older had seen a health care provider in the previous year; according to NYC HANES, approximately 4.7 million (75.1%) had. In both surveys, the in-care population was more likely to be older, female, non-Hispanic, and insured compared with the not-in-care population. The in-care population from the NYC HANES also had a higher prevalence of diabetes (16.7% vs 6.9%; *P* < .001), hypercholesterolemia (35.7% vs 22.3%; *P* < .001), and hypertension (35.5% vs 26.4%; *P* < .001) than the not-in-care population.

**Conclusion:**

Systematic differences between in-care and not-in-care populations warrant caution in using primary care data to generalize to the population at large. Future efforts to use primary care data for chronic disease surveillance need to consider the intended purpose of data collected in these systems as well as the characteristics of the population using primary care.

## Introduction

Widespread adoption of electronic health records (EHRs) in primary care practices has begun to transform the practice of medicine, with implications for patients and clinicians about the quality, continuity, and efficiency of care. Aside from their clinical utility, the richness of data in EHRs offers an opportunity to advance chronic disease surveillance through aggregating data (1). A major advantage of EHRs for this use over other data sources is that they can provide real-time data and clinically measured outcomes, which can complement data collected from traditional chronic disease surveillance methods, such as registries, surveys, and hospital discharge and medical claims databases (1). In the United States, national EHR incentive programs have catalyzed the transition from paper to electronic records and have led to a substantial volume of clinical data for public health research (2,3). By 2014, 83% of office-based primary care practices in the United States had adopted an EHR (4).

Although the uptake of EHRs in primary care practices presents a unique opportunity to leverage EHR data for chronic disease surveillance, the generalizability of these data for estimation of disease prevalence is of concern because some groups may be more likely or less likely than other groups to seek primary care. Predictors of primary care use tend to be female sex, higher educational attainment, older age, lower self-rated health status, increasing number of health problems, urban residence, US birth, and longer length of residence in new country if foreign born (5–9). Populations that may be underrepresented in EHR data might include healthy people who do not perceive a need for preventive care or people who are unable to access care (eg, the uninsured, undocumented immigrants) (10). The nonrandom missing data from the not-in-care population may bias estimates of disease prevalence and risk factors (11). Some EHR-based surveillance studies avoid this bias by generalizing their EHR-based surveillance data to the population in care (12,13), but others do not (14–19).

The objective of this study was to quantify hypothetical differences in the demographics and prevalence of risk factors and chronic diseases between in-care and not-in-care populations by using data from 2 population-based surveys of New York City residents. This study will help jurisdictions, including our own, determine whether to generalize their EHR-based prevalence estimates to the general public, which includes both in-care and not-in-care populations, or to generalize to the in-care population only.

## Methods

### Sample

We used data from the 2013 New York City Community Health Survey (CHS) and the 2013–2014 New York City Health and Nutrition Examination Survey (NYC HANES). The CHS is an annual telephone survey conducted by the New York City Department of Health and Mental Hygiene (DOHMH), modeled after the Behavioral Risk Factor Surveillance System, and targeted to noninstitutionalized adults (aged ≥18 y) with a cellular telephone or landline living in New York City (20). NYC HANES is a community-based examination survey modeled after the National Health and Nutrition Examination Survey, first conducted by DOHMH in 2004 and conducted again in 2013–2014 by the City University of New York School of Public Health and DOHMH jointly (21). Participants in NYC HANES were randomly selected noninstitutionalized adults (aged ≥20 y). For the CHS, data were restricted to participants aged 20 years or older with complete data on sex, age group, and neighborhood poverty; 576 respondents (6.5%) were excluded from the original sample because of these restrictions, and the resulting sample size was 8,131. No data restrictions were necessary for NYC HANES (n = 1,524).

### Measures

The in-care population was defined as people who saw a health care provider in the previous year. In the CHS, in-care was defined as an affirmative response to the following 2 questions: “Do you have one person or more than one person you think of as your personal doctor or health care provider?” and “Have you seen your personal doctor or health care provider in the last 12 months?” In NYC HANES, in-care was defined as a response of one or more to the question “During the past 12 months, how many times have you seen a doctor or other health care professional?” and an affirmative response to the question “Were any of these visits in the past 12 months at a doctor’s office or clinic for a checkup, advice about a health problem, or basic care?” Our sensitivity analysis included a variable to capture data on NYC HANES participants who had seen a health care provider from 1 to 3 years previously, defined as a response of “more than 1 year, but not more than 3 years ago” to the question “About how long has it been since you last saw or talked to a doctor or other health care professional about your health?”

Independent variables of interest were demographics, health care coverage and access, health indicators, and chronic conditions. Demographic variables were age, sex, race/ethnicity, marital status, neighborhood poverty, employment status, education, whether born in the United States (50 states and District of Columbia), years in the United States (if foreign born), and interview language. Neighborhood poverty was calculated as the percentage of population in the participant’s zip code living below 100% of the federal poverty level per the American Community Survey (ACS) 2008–2012 and was categorized as follows: less than 10% (low level of neighborhood poverty), 10% to 19%, 20% to 29%, and 30% to 100% (very high level of neighborhood poverty). Variables for health care coverage and access were having any health insurance, having Medicaid coverage (vs non-Medicaid coverage), and not obtaining needed medical care in the previous 12 months. Health indicators included self-rated health status, body mass index (BMI), smoking status, receiving an influenza vaccine in the previous 12 months, and receiving mental health treatment (medication or counseling) in the previous 12 months. BMI was based on self-reported height and weight in the CHS, and height and weight measurements were taken at the NYC HANES interview. We categorized BMI (kg/m^2^) as underweight (<18.5), normal (18.5–24.9), overweight (25.0–29.9), obese (30.0–39.9), or extremely obese (≥40.0). A current smoker was defined as having smoked 100 or more cigarettes in his or her lifetime and a response of “every day” or “some days” to a question about current smoking frequency. Chronic condition variables were based on history, ie, whether a participant had ever been told he or she had depression, diabetes, hypertension, or hypercholesterolemia by a health care provider. For hypercholesterolemia, data were restricted to women aged 45 or older and men aged 35 or older to mirror the age- and sex-targeted recommendations for routine hypercholesterolemia testing by the US Preventive Services Task Force. Nonspecific psychological distress was defined as a Kessler 6 (K6) score of 7 to 24 (22). In NYC HANES, we considered additional prevalence variables (ie, “gold standard” definitions) for diabetes (hemoglobin A1c ≥6.5, or ever told diabetes *and* currently taking diabetes medication), hypertension (blood pressure ≥140/90 mm Hg, or ever told hypertension *and* currently taking hypertension medication), and hypercholesterolemia (total cholesterol ≥240 mg/dL, or ever told hypercholesterolemia *and* currently taking cholesterol medication) because biomarkers and information on medications were available.

### Statistical analysis

We conducted bivariate analyses using Rao–Scott χ^2^ tests to compare indicators in the CHS and NYC HANES by in-care status. We also conducted 2 sensitivity analyses. The first sensitivity analysis compared NYC HANES participants classified as in-care (ie, seen a health care provider in the previous year) with participants who had last seen a health care provider from 1 to 3 years previously, to determine if we could generalize the in-care population to people with more remote health care contact. Having health insurance is a major determinant of seeking care, and with the Affordable Care Act (ACA), the number of Americans with health insurance increased (23). To determine how maximal uptake of health insurance under the ACA and subsequent care might affect the characteristics of the in-care population, we conducted a second sensitivity analysis. This analysis used NYC HANES data and χ^2^ tests to compare the demographics and health indicators of the in-care population and the uninsured not-in-care population. We also computed prevalence differences for health indicators between the in-care population and the uninsured not-in-care population combined with the in-care population.

All analyses were performed in SAS-callable SUDAAN (SAS version 9.2, SAS Institute; SUDAAN version 11.0.1, RTI International) to account for the complex sampling design. Estimates were weighted to the New York City population based on the ACS (2012 for CHS and 2013 for NYC HANES) and age-adjusted to the US 2000 standard population. Significance level was set at a 2-sided α of .05.

## Results

### CHS

According to the CHS, 71.7% (4,137,212) of adult New York City residents saw a health care provider in the previous year. We found significant differences in all demographic characteristics, insurance coverage, and health care access variables, except for Medicaid coverage, between the in-care and not-in-care populations (Table 1). In-care participants were more likely to be older (30.2% vs 10.6% for age ≥60 y), female (59.4% vs 42.0%), white non-Hispanic (39.5% vs 31.0%), married (44.3% vs 40.8%), born in the United States (51.7% vs 37.9%), residing longer in the United States if foreign born (76.8% vs 69.5% for ≥10 y), and having an English interview (80.6% vs 65.6%) than not-in-care participants. They were also more likely to reside in neighborhoods with the lowest levels of poverty (23.0% vs 17.2%), more likely to be unemployed or not in the labor force (41.8% vs 35.8%), a college graduate (36.4% vs 28.1%), and insured (90.9% vs 52.5%), and less likely to defer needed medical care (8.9% vs 17.0%) than not-in-care participants.

**Table 1:** Demographics, Health Care Coverage, and Access to Care Among New York City Adults in Care and Not in Care, 2013–2014^a^

Characteristic	2013 Community Health Survey			2013–2014 New York City Health and Nutrition Examination Survey (NYC HANES)				
	In Care Within Previous Year	Not in Care Within Previous Year	*P* b	In Care Within Previous Year	Not in Care Within Previous Year	*P* b ^, ^ c	In Care From 1 to 3 Years Previously^d^	*P* b ^, ^ e
**Sample size, n^f^**	6,166	1,921	—	1,135	386	—	200	—
**Weighted sample size, n**	4,137,212	1,639,236	—	4,701,244	1,584,505	—	796,727	—
**Proportion**	71.7 (70.3–73.0)	28.3 (27.0–29.7)	—	75.1 (72.2–77.8)	24.9 (22.2–27.8)	—	14.5 (12.4–16.8)^g^	—
**Age, y**								
20–39	32.9 (31.2–34.6)	60.8 (57.9–63.7)	<.001	36.3 (32.7–40.0)	58.6 (52.9–64.1)	<.001	64.1 (55.8–71.6)	<.001
40–59	36.9 (35.2–38.6)	28.6 (26.0–31.3)		36.6 (33.4–40.0)	30.2 (24.9–36.1)		25.2 (18.5–33.3)	
≥60	30.2 (28.7–31.7)	10.6 (8.9–12.6)		27.1 (23.9–30.5)	11.2 (7.8–15.7)		10.7 (6.6–17.0)	
**Sex**								
Female	59.4 (57.6- 61.2)	42.0 (38.8–45.3)	<.001	57.2 (54.3–60.1)	40.7 (35.3–46.3)	.001	39.6 (32.2–47.6)	<.001
Male	40.6 (38.8–42.4)	58.0 (54.7–61.2)		42.8 (39.9–45.7)	59.3 (53.7–64.7)		60.4 (52.4–67.8)	
**Race/ethnicity**								
White non-Hispanic	39.5 (37.8–41.2)	31.0 (28.0–34.2)	<.001	34.4 (28.9–40.3)	38.7 (31.0–47.1)	.03	45.5 (35.9–55.3)	.001
Black non-Hispanic	23.2 (21.7–24.7)	18.9 (16.5- 21.7)		23.2 (18.0–29.3)	15.7 (11.1–21.9)		16.3 (10.5–24.5)	
Hispanic	22.7 (21.3–24.1)	36.1 (33.0–39.3)		25.8 (21.7–30.4)	30.5 (23.5–38.5)		22.2 (16.0–29.9)	
Asian non-Hispanic^h^	12.4 (11.2–13.6)	12.3 (10.4–14.5)		13.8 (10.6–17.9)	13.1 (9.3–18.2)		15.1 (9.9–22.5)	
Other non-Hispanic^h^	2.3 (1.8–3.0)	1.6 (0.9–2.8)		2.8 (1.9–4.1)	1.8^i^ (1.0–3.5)		0.9^i^ (0.3–3.2)	
**Marital status**								
Married	44.3 (42.7–46.0)	40.8 (37.5–44.1)	<.001	44.1 (39.9–48.4)	40.8 (34.5–47.4)	<.001	36.5 (27.4–46.7)	<.001
Divorced or separated	12.6 (11.6–13.6)	14.1(12.0–16.6)		14.0 (12.0–16.3)	11.2 (7.7–16.2)		14.1 (8.1–23.3)	
Widowed	6.2 (5.6–7.0)	5.6 (4.1–7.6)		5.2 (4.1–6.5)	3.0^i^ (1.5–5.9)		1.1^i^ (0.3–4.5)	
Never married	30.4 (28.9–32.0)	29.7(27.4–32.1)		28.7(25.7–32.0)	35.0 (29.5–40.9)		38.1 (30.3–46.6)	
Living with partner	6.4 (5.5–7.5)	9.8 (8.3–11.6)		8.0 (6.3–10.1)	9.9 (7.0–13.9)		10.3 (7.0–14.8)	
**Neighborhood poverty level^j^**								
<10% (low)	23.0 (21.6–24.4)	17.2 (15.0–19.6)	<.001	23.7 (17.1–31.8)	21.3 (14.2–30.6)	.52	24.7^i^ (15.9–36.2)	.37
10%–19%	36.7 (35.0–38.5)	34.8 (31.7–38.0)		36.1 (28.1–45.0)	37.2 (27.8–47.6)		43.6^i^ (31.8–56.2)	
20%–29%	23.2 (21.7–24.7)	27.5 (24.6–30.6)		23.1 (16.5–31.4)	23.2 (15.2–33.7)		19.5 (11.9–30.2)	
30%–100% (very high)	17.1 (15.9–18.4)	20.5 (17.9–23.4)		17.1 (11.7–24.3)	18.3 (11.3–28.2)		12.2^i^ (6.5–21.8)	
**Employment status**								
Employed	58.2 (56.5–59.9)	64.2 (61.1–67.3)	<.001	59.0 (55.6–62.2)	64.4 (58.1–70.3)	.004	70.7 (62.4–77.9)	.001
Unemployed or not in labor force	41.8 (40.1–43.5)	35.8 (32.7–38.9)		41.0 (37.8–44.4)	35.6 (29.7–41.9)		29.3 (22.1–37.6)	
**Education**								
<High school	16.1 (14.8–17.5)	27.2 (24.2–30.5)	<.001	18.4 (15.6–21.6)	20.2 (14.5–27.5)	.84	14.1 (9.1–21.1)	.01
High school graduate or GED	23.6 (22.1–25.2)	24.0 (21.3–26.9)		24.0 (20.7–27.5)	24.2 (18.7–30.7)		23.6 (16.7–32.3)	
Some college or associate degree	23.9 (22.3–25.5)	20.7 (18.0–23.7)		22.9 (20.2–25.7)	22.0 (17.6–27.2)		19.5 (13.4–27.4)	
College graduate	36.4 (34.7–38.1)	28.1 (25.5–30.9)		34.8 (30.5–39.3)	33.5 (27.2–40.5)		42.8 (34.4–51.7)	
**US born**	51.7 (50.0–53.5)	37.9 (34.8–41.1)	<.001	49.5 (45.0–54.0)	48.6 (41.9–55.3)	.50	59.8 (50.7–68.2)	.002
**Length of residence in United States (if foreign born)**								
<5 y	11.1 (9.4–13.1)	16.4 (13.9–19.3)	<.001	10.7 (7.1–15.7)	11.5 (7.0–18.4)	.28	18.9^i^ (9.7–33.7)	.15
5–9 y	12.0 (10.3–14.0)	14.0 (11.3–17.3)		14.7 (11.6–18.4)	12.8 (8.5–18.9)		9.9^i^ (4.7–19.7)	
≥10 y	76.8 (74.5–79.0)	69.5 (65.8–73.1)		74.6 (69.9–78.9)	75.7 (68.4–81.7)		71.1^i^ (57.0–82.1)	
**Language of interview**								
English	80.6 (79.2–81.9)	65.6 (62.4–68.8)	<.001	84.6 (80.6–87.9)	80.4 (72.0–86.7)	.59	88.3 (80.2–93.3)	.10
Spanish	11.3 (10.2–12.4)	25.7 (22.8–28.7)		9.6 (7.2–12.7)	13.8 (8.4–22.0)		4.1^i^ (2.0–8.3)	
Russian	2.8 (2.3–3.4)	2.9 (1.9–4.4)		3.6^i^ (1.9–6.8)	3.7^i^ (1.4–9.5)		5.0^i^ (1.9–12.5)	
Chinese	5.3 (4.6–6.2)	5.8 (4.5–7.5)		1.8^i^ (0.9–3.5)	1.1^i^ (0.3–4.5)		1.6^i^ (0.2–10.3)	
Other	—	—		0.3^i^ (0.1–0.9)	1.0^i^ (0.2–3.8)		1.0^i^ (0.1–7.1)	
**No health insurance**	9.1 (7.9–10.4)	47.5 (44.1–50.8)	<.001	10.8 (8.9–13.0)	33.2 (28.3–38.4)	<.001	32.0 (24.5–40.5)	<.001
**Insurance type**								
Medicaid	26.9 (25.2–28.6)	28.1 (24.3–32.1)	.12	28.0 (23.7–32.6)	17.8 (13.1–23.6)	.005	18.1 (11.5–27.2)	.05
Non-Medicaid	73.1 (71.4–74.8)	71.9 (67.9–75.7)		72.0 (67.4–76.3)	82.2 (76.4–86.9)		81.9 (72.8–88.5)	
**Did not get needed medical care in previous 12 months**	8.9 (8.0–10.0)	17.0 (14.4–20.0)	<.001	8.7 (7.2–10.6)	10.6 (7.7–14.4)	.22	10.4 (6.8–15.5)	.32

Abbreviations: —, not applicable; GED, general educational development.

a All values are percentage (95% confidence interval) unless otherwise indicated. Column percentages may not add up to 100% because of rounding.

b All *P* values determined by Rao–Scott χ^2^ test.

c NYC HANES not in care within previous year vs NYC HANES in care within previous year.

d Defined as a response of “more than 1 year, but not more than 3 years ago” to the question “About how long has it been since you last saw or talked to a doctor or other health care professional about your health?”

e NYC HANES in care from 1 to 3 years previously vs NYC HANES in care within previous year.

f Sample sizes for the Community Health Survey in this row do not add to 8,131 (total sample size) because 44 participants did not respond (refused or responded with “don’t know”) to the in-care questions and were dropped from the analyses. For the NYC HANES, 3 participants did not respond, so sample sizes in this row do not add to 1,524 (total sample size).

g Proportion relative to the population in care within 3 years.

h Non-Hispanic Pacific Islanders are categorized as “Asian non-Hispanic” in the Community Health Survey, and “other non-Hispanic” in NYC HANES.

i Estimate should be interpreted with caution. Estimate’s relative standard error (a measure of estimate precision) is greater than 30%, the 95% confidence interval half-width is greater than 10, or the sample size is too small, making the estimate potentially unreliable.

j Percentage of population in the participant’s zip code living below 100% of the federal poverty level per the American Community Survey 2008–2012.

We also found significant differences in the health indicators and chronic conditions of the in-care and not-in-care populations (Table 2). The in-care population was more likely to have excellent (19.2% vs 17.8%) or very good (26.9% vs 22.9%) self-rated health and to be obese (20.6% vs 18.3%) or extremely obese (3.5% vs 2.9%). They were also less likely to be current smokers (14.9% vs 21.2%) and more likely to have received an influenza vaccine (47.3% vs 23.1%) and mental health treatment (14.2% vs 8.0%) in the previous 12 months. A significantly larger proportion of in-care participants had a history of diabetes (12.5% vs 5.4%), hypertension (31.6% vs 21.8%), hypercholesterolemia (39.6% vs 23.8%), and depression (16.4% vs 13.4%). In-care participants were less likely to have mild, moderate, or severe nonspecific psychological distress (K6 score 7–24; 20.3% vs 25.6%).

**Table 2:** Health Indicators and Chronic Conditions Among New York City Adults in Care and Not in Care, 2013–2014^a^

Indicator or Condition	2013 Community Health Survey			2013–2014 New York City Health and Nutrition Examination Survey (NYC HANES)				
	In Care Within Previous Year	Not in Care Within Previous Year	*P* b	In Care Within Previous Year	Not in Care Within Previous Year	*P* b ^, ^ c	In Care From 1 to 3 Years Previously^d^	*P* b ^, ^ e
**Sample size, n^f^**	6,166	1,921	—	1,135	386	—	200	—
**Weighted sample size, n**	4,137,212	1,639,236	—	4,701,244	1,584,505	—	796,727	—
**Proportion**	71.7 (70.3–73.0)	28.3 (27.0–29.7)	—	75.1 (72.2–77.8)	24.9 (22.2–27.8)	—	14.5 (12.4–16.8)^g^	—
**Self-rated health status**								
Excellent	19.2 (17.8–20.7)	17.8 (15.5–20.4)	.001	14.1 (11.5–17.2)	22.4 (18.0–27.5)	.001	18.2 (13.4–24.2)	.02
Very good	26.9 (25.2–28.5)	22.9 (20.3–25.6)		29.3 (26.7–32.0)	26.3 (21.1–32.1)		34.5 (25.9–44.3)	
Good	31.6 (29.9–33.3)	35.8 (32.8–38.9)		34.5 (31.2–37.9)	32.6 (26.5–39.4)		30.5 (22.9–39.2)	
Fair or poor	22.3 (21.0–23.7)	23.5 (20.6–26.7)		22.2 (19.3–25.3)	18.7 (13.8–24.9)		16.8 (10.5–25.8)	
**Body mass index^h^**								
Underweight	2.5 (1.9–3.2)	3.8 (2.6–5.5)	.007	2.0 (1.3–3.0)	2.3^i^ (1.0–5.3)	.10	3.1^i^ (1.0–9.3)	.06
Normal	40.6 (38.8–42.4)	38.7 (35.5–41.9)		32.2 (29.3–35.2)	33.4 (28.1–39.1)		36.3 (28.7–44.8)	
Overweight	32.8 (31.2–34.6)	36.3 (33.1–39.6)		34.6 (32.0–37.3)	36.4 (31.4–41.6)		35.5 (28.0–43.8)	
Obese	20.6 (19.1–22.1)	18.3 (15.7–21.3)		26.2 (23.4–29.1)	25.0 (19.9–31.0)		23.0 (16.4–31.2)	
Extremely obese	3.5 (2.9–4.2)	2.9 (2.0–4.3)		5.1 (3.8–6.8)	2.9^i^ (1.4–5.9)		2.1^i^ (0.7–6.2)	
**Smoking status^j^**								
Current	14.9 (13.6–16.3)	21.2 (18.6–24.1)	<.001	17.7 (15.1–20.8)	20.9 (15.9–27.0)	.27	20.2 (13.8–28.7)	.47
Former	20.7 (19.4–22.1)	18.1 (15.5–20.9)		21.6 (19.1–24.2)	23.6 (18.5–29.7)		30.0 (23.0–38.2)	
Never	64.4 (62.6–66.1)	60.7 (57.4–64.0)		60.7 (57.3–64.0)	55.4 (49.2–61.5)		49.7 (42.1–57.3)	
**Received influenza vaccine in previous 12 months**	47.3 (45.5–49.0)	23.1 (20.6–25.9)	<.001	47.6 (44.0–51.3)	23.3 (18.7–28.7)	<.001	23.0 (16.3–31.3)	<.001
**Mental health **								
Ever told depression	16.4 (15.1–17.9)	13.4 (11.0–16.2)	<.001	15.2 (13.0–17.7)	13.9 (10.2–18.7)	.21	10.8 (6.5–17.3)	.12
Nonspecific psychological distress (score on Kessler 6 scale [22])								
No or low (0–6)	79.7 (78.2–81.1)	74.4 (71.2–77.4)	.02	78.0 (74.7–81.0)	77.0 (71.5–81.7)	.63	76.8 (69.7–82.7)	.49
Mild or moderate (7–12)	15.1 (13.8–16.5)	19.0 (16.4–21.9)		17.2 (14.8–20.0)	17.2 (13.3–22.0)		17.3 (12.0–24.4)	
Severe (13–24)	5.2 (4.5–6.1)	6.6 (4.8–9.0)		4.8 (3.5–6.5)	5.8 (3.3–10.0)		5.8^i^ (3.0–11.2)	
Received mental health treatment in previous 12 months	14.2 (12.9–15.5)	8.0 (6.4–10.1)	<.001	19.2 (16.8–21.8)	11.4 (8.2–15.7)	<.001	9.3 (5.3–15.8)	<.001
**Diabetes**								
Ever told had diabetes	12.5 (11.5–13.5)	5.4 (4.0–7.3)	<.001	12.6 (10.6–14.8)	4.8^i^ (2.5–9.0)	<.001	2.5^i^ (0.7–8.8)	<.001
HbA1c ≥6.5 or ever told diabetes and takes medication	—	—	—	16.7 (14.3–19.3)	6.9 (4.1–11.3)	<.001	4.8^i^ (2.0–10.9)	<.001
**Hypertension **								
Ever told hypertension	31.6 (30.2–33.0)	21.8 (19.1–24.7)	<.001	32.5 (29.4–35.7)	16.2 (12.1–21.5)	<.001	15.2 (9.7–23.1)	<.001
Blood pressure ≥140/90 mm Hg or ever told hypertension and takes medication	—	—	—	35.5 (32.5–38.7)	26.4 (21.3–32.4)	<.001	28.2 (21.5–36.0)	<.001
**Hypercholesterolemia** k								
Ever told hypercholesterolemia	39.6 (35.9–43.5)	23.8 (20.6–27.3)	<.001	43.1 (36.0–50.5)	20.7 (14.8–28.1)	<.001	21.3 (13.1–32.9)	<.001
Total cholesterol ≥240 mg/dL or ever told hypercholesterolemia and takes medication	—	—	—	35.7 (28.6–43.5)	22.3 (14.4–32.8)	<.001	19.3^i^ (10.8–32.0)	.006

Abbreviations: —, not applicable; HbA1c, glycated hemoglobin.

a All values are percentage (95% confidence interval) unless otherwise indicated. Column percentages may not add up to 100% because of rounding.

b All *P* values determined by Rao–Scott χ^2^ test.

c NYC HANES not in care within previous year vs NYC HANES in care within previous year.

d Defined as a response of “more than 1 year, but not more than 3 years ago” to the question “About how long has it been since you last saw or talked to a doctor or other health care professional about your health?”

e NYC HANES in care from 1 to 3 years ago vs NYC HANES in care within previous year.

f Sample sizes for the Community Health Survey in this row do not add to 8,131 (total sample size) because 44 participants did not respond (refused or responded with “don’t know”) to the in-care questions and were dropped from the analyses. For the NYC HANES, 3 participants did not respond, so sample sizes in this row do not add to 1,524 (total sample size).

g Proportion relative to the population in care within 3 years.

h For CHS, based on self-reported height and weight; for NYC HANES, based on height and weight measurements taken at interview. Categorized (kg/m^2^) as underweight (<18.5), normal (18.5–24.9), overweight (25.0–29.9), obese (30.0–39.9), or extremely obese (≥40.0).

i Estimate should be interpreted with caution. Estimate’s relative standard error (a measure of estimate precision) is greater than 30%, the 95% confidence interval half-width is greater than 10, or the sample size is too small, making the estimate potentially unreliable.

j Current smoker defined as having smoked ≥100 cigarettes in his or her lifetime and a response of “every day” or “some days” to a question about the current smoking frequency.

k Data restricted to women aged ≥45 years and men aged ≥35 years.

### NYC HANES

According to the 2013–2014 NYC HANES, 75.1% (4,701,244) of adult New York City residents saw a health care provider in the previous year. We found significant differences in age, sex, race/ethnicity, marital status, employment status, health insurance status, and Medicaid coverage between the in-care and not-in-care populations (Table 1). Similar to the CHS in-care population, the NYC HANES in-care population was more likely to be older (27.1% vs 11.2% for age ≥60 y), female (57.2% vs 40.7%), married (44.1% vs 40.8%), unemployed or not in the labor force (41.0% vs 35.6%), and insured (89.2% vs 66.8%), but in contrast to the CHS, more likely to have Medicaid (28.0% vs 17.8%) than the not-in-care population.

We found significant differences between the 2 populations in health indicators (Table 2). The in-care population was more likely to have received an influenza vaccine (47.6% vs 23.3%) and mental health treatment (19.2% vs 11.4%) in the previous 12 months, and to have a history of diabetes (12.6% vs 4.8%), hypertension (32.5% vs 16.2%), or hypercholesterolemia (43.1% vs 20.7%). The populations did not significantly differ in self-rated health status, BMI, smoking status, depression, or nonspecific psychological distress; however, the distribution of these variables in NYC HANES was similar to their distribution in CHS. Additionally, the in-care population had a higher prevalence of diabetes (16.7% vs 6.9%), hypertension (35.5% vs 26.4%), and hypercholesterolemia (35.7% vs 22.3%).

In a comparison of NYC HANES participants who were in care from 1 to 3 years previously with participants in care within the previous year, we found significant differences in demographics (Table 1), health indicators, and chronic conditions (Table 2). The population in care from 1 to 3 years previously was more likely to be younger, male, non-Hispanic, unmarried, residing in neighborhoods with lower levels of poverty, employed, college graduates, born in the United States, and uninsured. Both populations significantly differed in health indicators and chronic conditions, and the variables for the population in care from 1 to 3 years previously were generally distributed similarly to those of the population not in care within the previous year.

In NYC HANES, 9.2% of the population reported being uninsured and not seeing any health care provider in the previous year. The demographic characteristics and health status of these people were significantly different from that of the in-care population. Compared with the in-care population, the uninsured not-in-care population was mostly younger than 60 years, male, white non-Hispanic, and living in poorer neighborhoods; had a lower prevalence of obesity, hypertension, hypercholesterolemia, and diabetes; and was less likely to have received an influenza vaccination in the previous 12 months. In a comparison of the in-care population and the uninsured not-in-care population combined with the in-care population, the prevalence estimates for most health indicators differed by no more than 1.0 percentage point, with the exception of influenza vaccination (−3.3 percentage points) and hypercholesterolemia (−9.4 percentage points) (Table 3).

**Table 3:** Health Indicators and Chronic Conditions Among the In-Care Population Combined With the Uninsured Not-in-Care Population in New York City, 2013–2014^a^

Indicator or Condition	Prevalence Estimate, %^b^ (95% CI)	Difference in Prevalence Between Existing In-Care Population and the In-Care Population Combined With the Uninsured Not-in-Care Population, Percentage Point
**Body mass index^c^**		
Underweight	1.8 (1.2–2.8)	−0.1
Normal	32.4 (29.6–35.4)	0.3
Overweight	35.1 (32.7–37.6)	0.5
Obese	26.1 (23.4–28.9)	−0.1
Extremely obese	4.6 (3.4–6.1)	−0.5
**Smoking status^d^**		
Current	18.5 (16.0–21.3)	0.8
Former	21.7 (19.3–24.4)	0.2
Never	59.7 (56.4–63.0)	−0.9
**Received influenza vaccine in previous 12 months**	44.3 (40.9–47.7)	−3.3
**Nonspecific psychological distress (score on Kessler 6 scale [** 22 **])**		
No or low (0–6)	77.8 (74.8–80.6)	−0.2
Mild or moderate (7–12)	17.6 (15.3–20.1)	0.3
Severe (13–24)	4.6 (3.4–6.2)	−0.2
**Blood pressure ≥140/90 mm Hg or ever told hypertension and takes medication**	34.6 (31.6–37.6)	−1.0
**Total cholesterol ≥240 mg/dL or ever told hypercholesterolemia and takes medication^e^**	26.3 (23.4–29.4)	−9.4
**HbA1c ≥6.5 or ever told diabetes and takes medication**	15.7 (13.4–18.4)	−1.0

Abbreviations: HbA1c, glycated hemoglobin; CI, confidence interval.

a Data source: 2013–2014 New York City Health and Nutrition Examination Survey (NYC HANES) (21).

b Column percentages may not add up to 100% because of rounding.

c Based on height and weight measurements taken at the NYC HANES interview. Categorized (kg/m^2^) as underweight (<18.5), normal (18.5–24.9), overweight (25.0–29.9), obese (30.0–39.9), or extremely obese (≥40.0).

d Current smoker defined as having smoked ≥100 cigarettes in his or her lifetime and a response of “every day” or “some days” to a question about the current smoking frequency.

e Data restricted to women aged ≥45 years and men aged ≥35 years.

## Discussion

We identified substantial differences between the in-care population and not-in-care population of New York City. In both surveys, the in-care population was disproportionately older, female, non-Hispanic, married, out of the labor force, more educated, insured, and not living in poor neighborhoods. The in-care population also had a higher prevalence of chronic diseases and obesity but was less likely to smoke than the not-in-care population.

These findings support our hypothesis that the in-care and not-in-care populations in New York City have systematic demographic differences, and the in-care population is sicker. Our findings on differences in age, sex, marital status, and smoking status are consistent with studies conducted outside of New York City (7,8,24). Although some EHR-based surveillance studies have not generalized their data to an in-care population (14–19), our results suggest that at least in New York City and perhaps in other jurisdictions, the in-care population is the most appropriate population for generalizing EHR estimates because of the differences between the in-care and not-in-care populations.

In a comparison of findings from the CHS and NYC HANES, some variables were significantly different between populations in one survey but not in the other; however, the directionality and magnitude were similar for most of these variables. Some of the observed differences were probably attributable to differences in sample size between the 2 surveys (n = 1,524 in NYC HANES vs n = 8,131 in CHS), but there may also be real differences in sample characteristics attributable to differences in sampling frames (random-digit–dialed vs address-based), incentives and barriers to participation (financial compensation and specimen collection in NYC HANES), interview mode (telephone vs in-person), or the wording of the questions used to classify the in-care population.

Our first sensitivity analysis revealed differences between people who saw a health care provider within the previous year and those who had their last health care contact from 1 to 3 years previously. The latter were generally more similar to the not-in-care population than they were to the population in care within the previous year, with the exception of more likely being non-Hispanic and born in the United States. This difference is important to consider not only for defining the optimal population for generalizing EHR estimates but also for defining patient inclusion criteria for the EHR cohort. These findings support the concept that the length of time since the most recent visit of patients sampled should parallel the definition of the population to which the prevalence estimates are generalized.

Our second sensitivity analysis assessed how maximal insurance uptake and health care utilization under the ACA might change the in-care population in New York City. A lower level of chronic disease was observed in the uninsured not-in-care population, and if this small group of people were to become insured and seek care, we would expect a minimal decline in the prevalence estimates of the in-care population of New York City. However, the results should be interpreted with caution because many possible reasons exist for why the eligible uninsured may not seek insurance under the ACA or why people do not seek care even if insured. Furthermore, these findings may vary by jurisdiction, depending on whether there is Medicaid expansion or not.

A major strength of this study was our use of data from 2 surveys that represented New York City’s diverse adult population. Many of the same questions were asked in both surveys, allowing us to see how different survey methodologies may have influenced our results. Furthermore, because self-reported data are subject to bias (eg, recall, social desirability), the use of biomarkers in NYC HANES allowed us to objectively characterize BMI and chronic health conditions of the in-care and not-in-care populations, increasing confidence in our findings. Although the focus of our study was to inform the generalizability of EHR-based prevalence estimates, our data also offer important insights into urban health status and unmet need for primary care among people with chronic conditions. Nevertheless, our study has some limitations. The in-care populations examined in this study might have included people seeking primary care from nontypical primary care settings (ie, specialists), and our findings may be specific to the United States (or New York City) only.

The differences observed between the in-care and not-in-care adult populations of New York City in this study confirmed our preliminary decision to limit generalization of prevalence estimates generated by the NYC Macroscope to the in-care population of New York City. (The NYC Macroscope is a surveillance system that uses EHRs to track chronic conditions managed by primary care practices [www.nyc.gov/html/doh/html/data/nycmacroscope.shtml]). Consequently, we are using data on age, sex, and neighborhood poverty distribution from the CHS in-care population to weight NYC Macroscope estimates. We validated 2013 NYC Macroscope prevalence estimates of smoking, obesity, depression, and influenza vaccination as well as data on the prevalence, treatment, and control of diabetes, hypertension, and hypercholesterolemia against in-care population estimates from the CHS and NYC HANES (25).

This study found significant differences between the in-care and not-in-care populations in New York City. Surveillance systems that use EHRs from primary care practices for monitoring chronic diseases should consider the intended purpose of the data collected and the systematic differences between in-care and not-in-care populations in the generalization of results.
