# Erratum, Vol. 13, April 28 Release

**DOI:** 10.5888/pcd14.150500e

**Published:** 2017-04-13

Because of an author error in data checking, incorrect prevalence estimates were reported for self-rated health status in an article on the 2013–2014 New York City Health and Nutrition Examination Survey, “Characterizing Adults Receiving Primary Medical Care in New York City: Implications for Using Electronic Health Records for Chronic Disease Surveillance.”

Two sentences in the fourth paragraph of the Results section were changed accordingly, and the paragraph now reads as follows: “We found significant differences between the 2 populations in health indicators (Table 2). The in-care population was less likely to report excellent health (14.1% vs 22.4%), more likely to have received an influenza vaccine (47.6% vs 23.3%) and mental health treatment (19.2% vs 11.4%) in the previous 12 months, and more likely to have a history of diabetes (12.6% vs 4.8%), hypertension (32.5% vs 16.2%), or hypercholesterolemia (43.1% vs 20.7%). The populations did not significantly differ in BMI, smoking status, depression, or nonspecific psychological distress; however, the distribution of these variables in NYC HANES was similar to their distribution in CHS. Additionally, the in-care population had a higher prevalence of diabetes (16.7% vs 6.9%), hypertension (35.5% vs 26.4%), and hypercholesterolemia (35.7% vs 22.3%).”

In addition, the values for self-rated health status were corrected in Table 2. The article was corrected on March 28, 2017, and appears online at https://www.cdc.gov/pcd/issues/2016/15_0500.htm. We regret any confusion or inconvenience this error may have caused.

